# Frequency and clinical features of hearing loss caused by *STRC* deletions

**DOI:** 10.1038/s41598-019-40586-7

**Published:** 2019-03-13

**Authors:** Yoh Yokota, Hideaki Moteki, Shin-ya Nishio, Tomomi Yamaguchi, Keiko Wakui, Yumiko Kobayashi, Kenji Ohyama, Hiromitsu Miyazaki, Rina Matsuoka, Satoko Abe, Kozo Kumakawa, Masahiro Takahashi, Hirofumi Sakaguchi, Natsumi Uehara, Takashi Ishino, Tomoki Kosho, Yoshimitsu Fukushima, Shin-ichi Usami

**Affiliations:** 1 Department of Otorhinolaryngology, Shinshu University School of Medicine, Matsumoto, Japan; 2 Department of Hearing Implant Sciences, Shinshu University School of Medicine, Matsumoto, Japan; 3 Department of Medical Genetics, Shinshu University School of Medicine, Matsumoto, Japan; 4 Center for Medical Genetics, Shinshu University Hospital, Matsumoto, Japan; 5 Department of Otolaryngology-Head & Neck Surgery, Iwate Medical University, Morioka, Japan; 6 Department of Otorhinolaryngology, Tohoku Rosai Hospital, Sendai, Japan; 7 Department of Otorhinolaryngology-Head and Neck Surgery, Tohoku University School of Medicine, Sendai, Japan; 8 Department of Otorhinolaryngology, Juntendo University Faculty of Medicine, Tokyo, Japan; 9 Department of Otorhinolaryngology, Toranomon Hospital, Tokyo, Japan; 10 Department of Otorhinolaryngology, Head and Neck Surgery, Yokohama City University School of Medicine, Yokohama, Japan; 11 Department of Otorhinolaryngology, International University of Health and Welfare, Mita Hospital, Tokyo, Japan; 12 Department of Otorhinolaryngology-Head and Neck Surgery, Kyoto Prefectural University of Medicine, Kyoto, Japan; 13 Department of Otolaryngology-Head and Neck Surgery, Kobe University School of Medicine, Kobe, Japan; 14 Department of Otorhinolaryngology, Head and Neck Surgery, Graduate School of Biomedical and Health Sciences, Hiroshima University, Hiroshima, Japan; 15 Research Center for Support to Advanced Science, Shinshu University, Matsumoto, Japan; 16 Shinshu University School of Medicine, Matsumoto, Japan

## Abstract

Sensorineural hearing loss is a common deficit and mainly occurs due to genetic factors. Recently, copy number variants (CNVs) in the *STRC* gene have also been recognized as a major cause of genetic hearing loss. We investigated the frequency of *STRC* deletions in the Japanese population and the characteristics of associated hearing loss. For CNV analysis, we employed a specialized method of Ion AmpliSeq^TM^ sequencing, and confirmed the CNV results via custom array comparative genomic hybridization. We identified 17 probands with *STRC* homozygous deletions. The prevalence of *STRC* homozygous deletions was 1.7% in the hearing loss population overall, and 4.3% among mild-to-moderate hearing loss patients. A 2.63% carrier deletion rate was identified in both the hearing loss and the control population with normal hearing. In conclusion, our results show that *STRC* deletions are the second most common cause of mild-to-moderate hearing loss after the *GJB2* gene, which accounts for the majority of genetic hearing loss. The phenotype of hearing loss is congenital and appears to be moderate, and is most likely to be stable without deterioration even after the age of 50. The present study highlights the importance of the *STRC* gene as a major cause of mild-to-moderate hearing loss.

## Introduction

Sensorineural hearing loss (SNHL) is common deficit at birth, affecting approximately 2 in 1,000 births^1^. Genetic factors account for 50–70% of SNHL. The major form of inheritance is autosomal recessive, which accounts for 75% of cases. Approximately 100 genes have been recognized as causative factors for SNHL, with the majority of causative alterations in the genes being single nucleotide variants (SNVs) or small insertions/deletions (indels). Recently, copy number variants (CNVs) have also been found to play an important role in many human diseases including neural developmental disorders^2,3^. CNVs; i.e., alterations through the deletion, insertion, or duplication of approximately 1 kb or more of a gene, are thought to affect gene expression, variation in phenotype, and adaptation via gene disruption, which may impact disease traits. More recently, CNVs have been recognized as a major cause of SNHL. Shearer *et al*. reported that CNVs were identified in 16 of 89 hearing loss-associated genes, with the *STRC* gene being the most common cause of SNHL^4^.

The *STRC* gene is a known deafness-associated gene causing mild-to-moderate hearing loss, and is a part of a large deletion in chromosome 15q15.3 at the DFNB16 locus. The incidence of *STRC* deletions has been estimated to be between 1% and 5% in deaf populations in previous reports^4–8^. However, there have been only a few reports on hearing loss resulting from CNVs including those in the *STRC* gene. The interpretation of sequence data of the *STRC* gene is challenging due to the existence of the pseudo-*STRC* gene (p*STRC*), which has 98% homology to the functional *STRC* gene. The p*STRC* arose in a segmental duplication with other genes (e.g., *CATSPER*2 located 100 kb downstream in chromosome 15q15.3); therefore, it is thought to be difficult to detect SNVs or CNVs in this region. Further, the clinical importance of the chromosomal deletion in the region harboring *STRC* to *CATSPER2* is associated with not only hearing loss but also infertility in males, referred to as deafness infertility syndrome, due to the fact that *CATSPER2* plays essential roles in sperm motility^9–11^.

Currently, massively parallel next generation sequencing (NGS) is being widely applied to genetic testing in clinical settings, and is able to allow precise diagnosis through data annotation with specific bioinformatics pipelines. In Japan, genetic testing for SNHL using NGS has been approved for coverage by social health insurance, and is currently being performed in clinical settings^12^. However, CNV analysis with the NGS dataset remains challenging, and it is thought to be difficult to detect CNVs accurately, especially in case of targeted NGS for hearing loss genes alone.

In the present study, we used the NGS platform to analyze CNVs in the *STRC* gene, and confirmed their existence via high-resolution array genomic hybridization (aCGH) analysis of the entire *STRC* genomic region. The aims of the study were to estimate the prevalence of CNVs in the *STRC* region in a Japanese deaf population, and obtain a more precise characterization of the clinical features.

## Subjects and Methods

### Ethics approval

All procedures were approved by the Shinshu University Ethical Committee as well as the respective ethical committees of the other participating institutions (Approval number: 576). All methods were in accordance with the Shinshu University Ethical Committee for Human Genetic Research guidelines and regulations. Informed consent was obtained from all subjects or parents of the proband for participation in the study.

### Subjects

One thousand twenty-five (1,025) Japanese subjects (age range, 0–70 years; mean, 11.8 years) from unrelated and non-consanguineous families were identified from 67 otolaryngology clinics in 28 prefectures across Japan between February 2012 and October 2015. All subjects had presumed non-syndromic bilateral SNHL. Clinical information and blood samples were obtained for each proband and for all consenting affected and unaffected relatives. Collected data included (1) pure-tone audiograms, and behavioral audiometry or auditory brain stem responses (ABR); (2) medical history, including onset of hearing loss, and progression of hearing loss; and 3) temporal bone imaging (computed tomography and/or magnetic resonance) if done. Hearing levels were classified based on the better hearing ear as normal, <20 dB; mild hearing loss, 21–40 dB; moderate hearing loss, 41–70 dB; severe hearing loss, 71–95 dB; and profound hearing loss, >95 dB. To clarify the prevalence of *STRC* deletions in the normal hearing population, we examined 152 normal control individuals using the same CNV analysis with the NGS dataset as used for the hearing loss subjects. The age range of each individual was 20–30 years. Ear examination and pure-tone audiometry were performed and showed normal results.

## Methods

### Amplicon resequencing with NGS

Amplicon libraries were prepared using an Ion AmpliSeq^TM^ Custom Panel (Applied Biosystems, Life Technologies), per with the manufacturer’s instructions, for 68 genes reported to cause non-syndromic hereditary HL (the list of genes is shown in Supplementary Table S1). The detailed sample preparation protocol has been described elsewhere^13^. We analyzed 45 samples in one Ion AmpliSeq^TM^ sequencing reaction to set 45 samples as a one-batch reaction. Sequencing was performed following the manufacturer’s instructions. NGS was performed with an Ion Torrent Personal Genome Machine system using an Ion PGM^TM^ 200 Sequencing Kit and an Ion 318^TM^ Chip (Life Technologies). The sequence data were mapped against the human genome sequence (build GRCh37/ hg19) with a Torrent Mapping Alignment Program. After sequence mapping, the DNA variant regions were piled up using the Torrent Variant Caller plug-in software. After variant detection, their effects were analyzed using ANNOVAR software.

### Copy number analysis in the NGS dataset

We employed our recently published specialized method for Ion AmpliSeq^TM^ sequencing that utilizes multiplex PCR-based targeted genome enrichment^14^. The depth of coverage information for each amplicon included in the barcode/amplicon coverage matrix file was used for copy number analysis. Normalization of coverage data depth was performed after considering the relative value of each amplicon. After normalization and removal of outlier data, the normalized relative read depths of amplicons were sorted based on chromosome position order and separated based on chromosome and gene identity, which were then plotted as a graph using conventional spread sheet software (Microsoft Excel^TM^) (Supplementary Fig. S1). All CNVs in the *STRC* region were curated through manual inspection from the graph.

### Copy number analysis via custom array-CGH

The commercial whole-genome array-CGH provided only one probe for every 3,000–8,000 bp, and only a few probes lay across the *STRC* genomic region (Supplementary Fig. S2). Therefore, we designed the custom aCGH for 68 known deafness-associated genes using the Agilent database (Agilent SureDesign, Agilent Technologies, Santa Clara, CA), and the probes which lay across specific chromosomal regions of those genes at 150–200 bp intervals as a design-setting on the Agilent 8 × 60 K platform (Agilent Technologies, Santa Clara, CA)^15^. There were 535 probes laid across the *STRC* and *CATSPER2* region (chr15:43600000–44330000), and probe intervals varied depend on the design platform due to highly homology of pseudogenes. We used the same DNA samples as used for amplicon resequencing, and quality assessment was also performed. Ten microliters of genomic DNA solution (0.5 µg of DNA) were fragmented, labeled with cyanine-3 for reference DNA samples and cyanine-5 for subjects, and then hybridized. Scanning of the array was carried out per the manufacturer’s recommended protocols. Scanned aCGH data were analyzed using CytoGenomics software version 3.0.6.6 (Agilent Technologies) (Supplementary Fig. S1).

### Ethics approval and consent to participate

All procedures were approved by the Shinshu University Ethical Committee as well as the respective ethical committees of the other participating institutions. Informed consent was obtained from all subjects or parents of the proband for participation in this study, which was approved by the human subjects’ ethical committee of each institution.

## Results

### Clinical characteristics of hearing loss patients and detected CNVs

As shown in Table 1, of the 1,025 subjects (age range, 0–70 years, mean age, 11.8 years), the distribution of SNHL inheritance mode was 264, 723, and 38 for segregating autosomal dominant, autosomal recessive or sporadic, and unknown, respectively. When classified based on age of onset as congenital–6 years, 7–18 years, adulthood (>18 years old), or unknown, most of the subjects with a causative *STRC* deletion were diagnosed with SNHL by adolescence. We found causative homozygous *STRC* deletions in 14 of the 723 cases categorized as segregating autosomal recessive or sporadic (1.94%), and in 3 of the 264 cases with autosomal dominant inheritance (1.14%). We identified duplications (3 copies) of *STRC* in 19 subjects (1.85%). It was unclear whether the 3 *STRC* copies were pathogenic or had any impact on phenotypes. We also found 27 subjects with *STRC* heterozygous deletions defined as carrier deletions. The frequency of carrier *STRC* deletions was 2.63% (27/1,025) in the hearing loss cohort, which was identical (2.63%, 4/152) to that in the normal hearing controls.

**Table 1:** Clinical characteristics of hearing loss patients and detected copy number variants.

		Total number	2-copy loss (%)	1-copy loss (%)	3-copy (%)
All cases		1025	17 (1.66)	27 (2.63)	19 (1.85)
Inheritance	Dominant	264	3 (1.14)	7 (2.65)	1 (0.38)
	Recessive/Sporadic	723	14 (1.94)	18 (2.49)	17 (2.35)
	Unknown	38	0 (0)	2 (5.26)	1 (2.63)
Age of onset	Congenital–6 years	625	15 (2.40)	16 (2.56)	14 (2.24)
	7–18 years	115	2 (1.74)	3 (2.61)	1 (0.87)
	Adult (>18 years)	264	0 (0)	8 (3.03)	3 (1.14)
	Unknown	21	0 (0)	0 (0)	1 (4.76)
Type of hearing loss	Mild-moderate	398	17 (4.27)	17 (4.27)	2 (0.50)
	Severe-profound	516	0 (0)	9 (1.74)	17 (3.29)
	Asymmetry	111	0 (0)	1 (0.91)	0 (0)
Normal hearing controls		152	0 (0)	4 (2.63)	3 (1.97)

Mild-to-moderate hearing loss is defined as >20 dB, = < 70 dB, bilateral, and interaural threshold gap < 15 dB. Severe-to-profound hearing loss is defined as >70 dB on both sides. Asymmetric hearing loss is defined as better hearing = < 70 dB and poorer hearing >70 dB, or interaural threshold gap >15 dB.

### Prevalence of CNVs in STRC among the subjects diagnosed with genetic hearing loss

**Figure 1:**
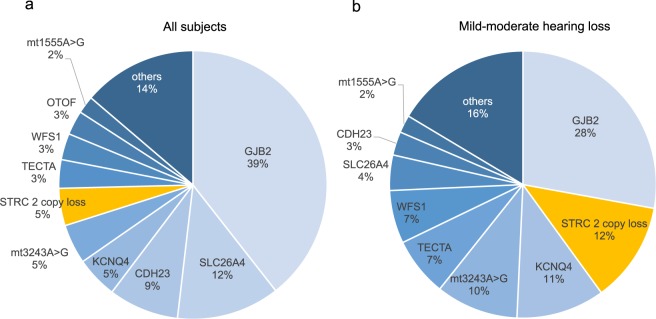
Prevalence of CNVs in *STRC*. Among the 1,025 subjects, we identified genetic causes in 395 probands (diagnosis rate, 38%), with variants in *GJB2 being* the most commonly identified (39%). CNVs implicated in *STRC* homozygous deletions (2 copies) accounted for 5% (17/395) of all subjects (**a**). Prevalence of *STRC* homozygous deletions was 12% (17/140) in subjects with mild-to-moderate SNHL (**b**).

As shown in Fig. 1, of the 1,025 subjects, the genetic causes of SNHLs were identified in 395 probands (diagnosis rate, 38%), with SNVs in *GJB2* being the most commonly identified (39%) among all subjects across all forms of inheritance. CNVs implicated in homozygous deletions (2 copies) in *STRC* accounted for 5% (17/395) of all subjects (Fig. 1a), whereas when classified based on hearing level as mild-to-moderate or severe-to-profound, the prevalence of causative *STRC* deletions was 12% (17/140) in the subjects with mild-to-moderate SNHL (Fig. 1b). Consequently, CNVs in *STRC* were the second most common cause of mild-to-moderate SNHL after SNVs in *GJB2*. None of the subjects with severe-to-profound or asymmetric SNHL had disease-causing CNVs in *STRC* (Table 1).

### Clinical features of STRC homozygous deletions

**Figure 2:**
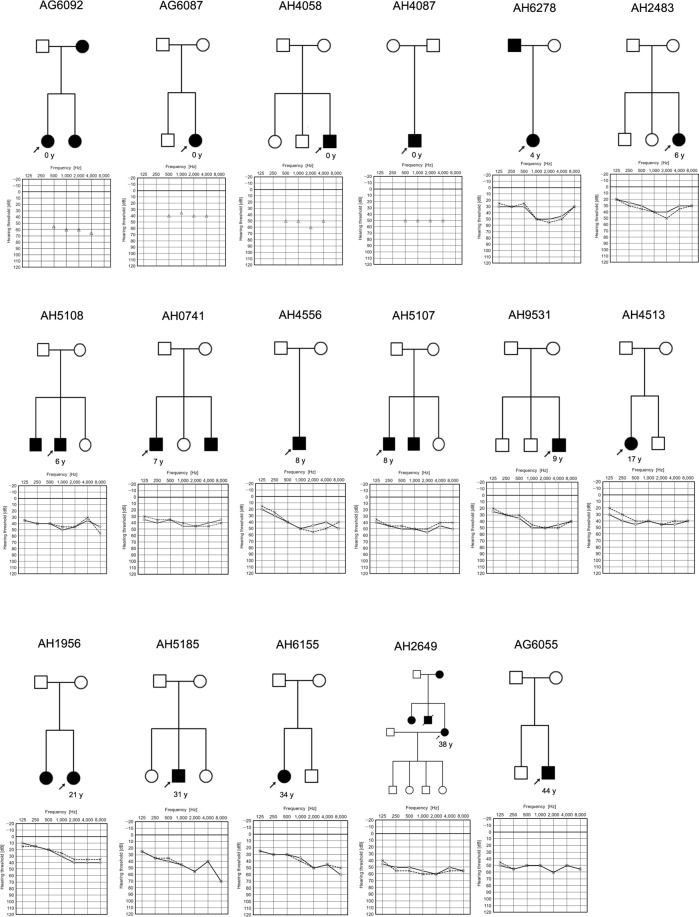
Pedigree and audiograms of each family with a homozygous *STRC* deletion. The age noted in the pedigree was the age at which the audiogram was obtained.

**Figure 3:**
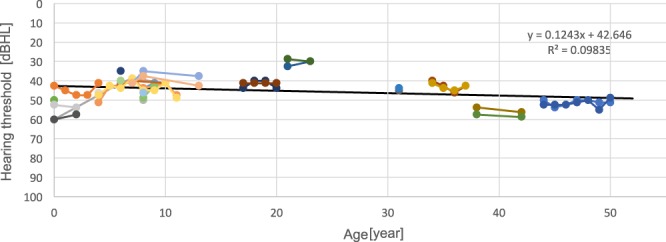
Hearing levels of nine subjects obtained from serial audiograms. Each connected line indicates the variation in the respective subject from 0 to 50 years of age. The hearing levels showed a slight decline (y = 0.12x), and hearing deterioration was likely aged-related.

As shown in Table 2, of the 17 subjects with *STRC* homozygous deletions, 7 individuals had undergone newborn hearing screening (NHS) and all had failed. Among the other individuals, hearing loss was identified at wellness checkups during elementary school years, or they became aware of hearing loss around the ages of 6 to 12. A distortion product otoacoustic emission (DPOAE, a tool for assessing cochlear status, specifically hair cell function) test has been undertaken by several individuals, and showed an absence of response indicating SNHL. The average hearing levels ranged from 28.8 to 60.8 dB, although the age at which the audiogram was taken varied (Fig. 2). Therefore, we assessed the deterioration of hearing in each individual by means of serial audiograms that were obtained every year for nine patients (Fig. 3). Figure 3 shows that hearing loss among these patients appeared to remain around the mild-to-moderate level, even in their fifties, and appeared not to deteriorate. The hearing levels showed a slight decline (y = 0.12x) and hearing deterioration was likely age-related, suggesting that it was not affected by the *STRC* deletion alone.

**Table 2:** Summary of clinical features of subjects affected with *STRC* homozygous deletions.

ID	Heredity	Sex	Age at audiogram	Average hearing levels	DPOAE	Awareness
AG6092	AD	F	3 m	60.8	na	NHS
AG6087	AR/Spo	F	4 m	42.5	No response	NHS
AH4058	AR/Spo	M	4 m	52.5	na	NHS
AH4087	AR/Spo	M	6 m	50.0	na	NHS
AH6278	AD	F	4 y	47.5	na	Medical checkup
AH2483	AR/Spo	F	6 y	35.0	na	Medical checkup
AH5108	AR/Spo	M	6 y	40.0	na	NHS
AH0741	AR/Spo	M	7 y	41.3	na	Medical checkup
AH4556	AR/Spo	M	8 y	43.8	na	Medical checkup
AH5107	AR/Spo	M	8 y	46.3	No response	NHS
AH9531	AR/Spo	M	9 y	43.8	na	NHS
AH4513	AR/Spo	F	17 y	41.3	No response	Elementary school days
AH1956	AR/Spo	F	21 y	28.8	No response	Elementary school days
AH5185	AR/Spo	M	31 y	43.8	na	Elementary school days
AH6155	AR/Spo	F	34 y	40.0	na	Elementary school days
AH2649	AD	F	38 y	53.8	na	Junior high school days
AG6055	AR/Spo	M	44 y	50.0	No response	Elementary school days

Abbreviations: AD, autosomal dominant; AR/Spo, autosomal recessive/sporadic; DPOAE, distortion product otoacoustic emission; NHS, newborn hearing screening.

### Confirmation of the STRC genomic region via array CGH

We performed array CGH for 17 probands in whom homozygous deletions in the *STRC* region were detected using NGS data. Fifteen cases had homozygous long deletions containing both *STRC* and *CATSPER2* genes. Two cases (AG6087 and AH5185) showed homozygous deletions in the *STRC* gene, not including the *CATSPER2* gene.

Supplementary Fig. S1 shows an enlarged view of the homozygous *STRC* and *CATSPER2* deletions detected using array CGH analysis.

## Discussion

In the present study, we identified homozygous deletions in the *STRC* genomic region leading to SNHL in 17 of 1,025 Japanese deafness cases (1.66%; 17/1,025), and heterozygous deletions in 27 subjects (2.63%), indicating that the frequency of CNVs in this region was 4.29% (44/1025). Francey *et al*. studied 659 probands with bilateral SNHL and identified 7 homozygous and 10 heterozygous deletions in the *STRC* region using SNP genotyping array; among 10 probands with heterozygous deletions, SNVs or interstitial deletions were identified on the trans-allele in 4 probands, which were defined as compound heterozygotes of CNV and SNV resulting in hearing loss^5^. Shearer *et al*. identified causative mutations in *STRC* including homozygous deletions, gene conversions, and heterozygous deletions in-trans leading to missense changes, in 37 out of 686 hearing loss patients (5.4%) through analysis of NGS read data in CNV detection^4^. Vona *et al*. also reported homozygous or heterozygous *STRC* deletions as a cause of hearing loss in 9 probands among 94 SNHL probands using whole-genome aCGH^7^; among 9 probands with heterozygous deletions, SNVs were identified on the trans-allele in 4 probands. In the present study, 17 out of 27 *STRC* deletion carriers exhibited mild-to-moderate hearing loss. We inferred that 17 carriers might have a causative SNV on the other allele in the *STRC*, suggesting the presence of SNV and CNV as a compound heterozygote. A limitation of our study was that we focused on identification of CNVs alone and detected gene gains or deletions in the *STRC* genomic region; indeed, it was likely to be more challenging to detect SNVs due to low reliability of NGS read data of the region with highly homologous pseudogenes.

The prevalence of CNVs in the *STRC* region might vary among different populations, or the results could be influenced by the different CNV detection methods used such as SNP array or NGS. In the present study, we undertook CNV analysis using NGS read data as a first screening step followed by confirmation with aCGH, which is thought to be a robust method for CNV detection. Whole-genome array CGH designs are based on principles described in several publications and cover targeted regions including individual disease-related genes. For the *STRC* gene and its surrounding region, only a few probes lay across the whole-genome CGH array. We therefore designed a custom array CGH with a high density of probes for deafness-associated genes including the *STRC* genomic region. We found a false-positive result in only one sample in which CNV analysis with NGS read data showed a homozygous deletion, while aCGH revealed a normal copy number. Therefore, our present findings regarding the frequency of CNVs in the *STRC* region in the largest cohort studied to-date, while preliminary, are thought likely to be accurate for subjects with SNHL. We were able to identify gene deletions or duplications in the *STRC* region, but could not detect gene conversions or balanced rearrangement. Thus, the frequency of *STRC* variations causing SNHL is expected to be much higher if conversions could be detected.

The frequency of carrier *STRC* deletions was 2.63% (4/152) in the normal hearing controls, which was identical (2.63%, 27/1,025) to that in the hearing loss cohort. The frequency of carrier mutations in the *GJB2* gene, which is the most prevalent cause of autosomal recessive deafness, is reported to be approximately 2% in the normal hearing population^16^. Although the frequency of heterozygous *GJB2* mutations is similar to that of heterozygous *STRC* deletions, the frequency of hearing loss due to homozygous *STRC* deletions is reported to be much lower than that due to homozygous/compound heterozygous *GJB2* mutations. One possible reason for this discrepancy is that the detection method using NGS dataset and custom aCGH employed in this study was fundamentally challenging; however, the observed deletions were reliable. The basis for the relationship between carrier frequency and prevalence of hearing loss with *STRC* deletions is currently unclear.

**Figure 4:**
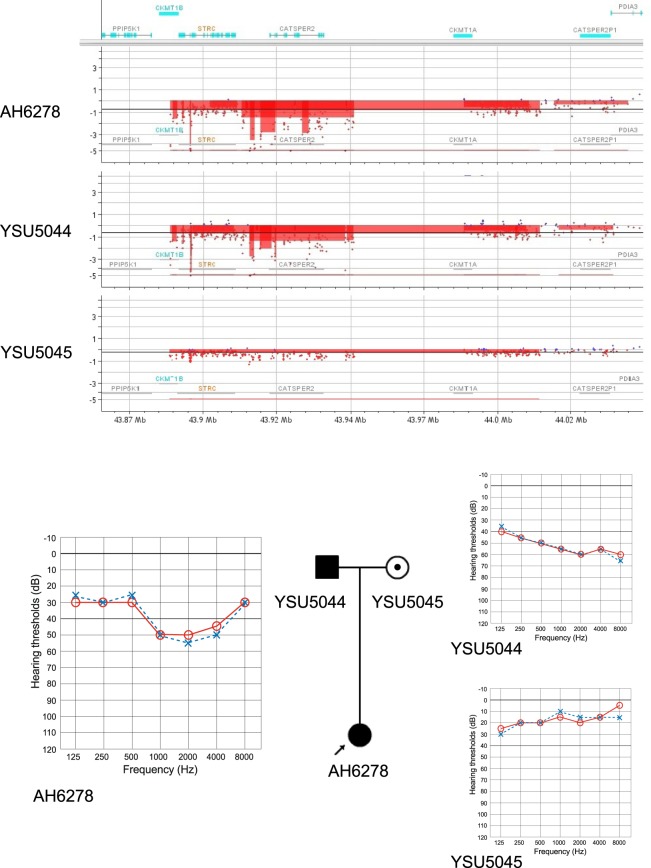
Results of custom array comparative genomic hybridization (aCGH) for the proband AH6278 and her parents. The upper panel shows a homozygous chromosomal deletion in the region containing *STRC* to *CATSPER2* in AH6278 (proband) and YSU5044 (father), and a heterozygous deletion in the *pSTRC* region in YSU5045 (mother) identified using CytoGenomics software. The lower panel shows audiograms and the pedigree, indicating pseudodominant inheritance.

Interestingly, we found causative homozygous *STRC* deletions in 3 probands (17.6%) with autosomal dominant-appearing inheritance among the 17 probands showing all forms of inheritance, indicating pseudodominant inheritance. As shown in Fig. 4, the proband AH6278 had homozygous deletions in *STRC*, and segregation analysis via aCGH showed that her father (YSU5044) had a homozygous deletion and that her mother (YSU5045) was a heterozygous carrier. Due to higher carrier frequency of deletions in the *STRC* region (2.63%; 27/1,025), it was possible to identify homozygous deletions in *STRC* even in families showing autosomal dominant inheritance. On the other hand, in our cohort, mutations in the *GJB2* gene, which is the most prevalent cause of autosomal recessive deafness, were found to follow pseudodominant inheritance in 15 (10.1%) of 149 probands showing all forms of inheritance. The carrier frequency of the SNVs in *GJB2* was previously reported to be 6.7% in Japanese bilateral SNHL populations^16^.

Approximately 70% of hereditary hearing loss patients exhibit autosomal recessive inheritance and are affected with severe-to-profound congenital SNHL. The major causative genes with recessive inheritance are *GJB2*, *SLC26A4*, and *CDH23*, which cause severe-to-profound congenital SNHL^12,17^. Most of the mutations in these genes are SNVs or small insertions/deletions (indels). The major genetic causes of mild-to-moderate SNHL in recessive inheritance remain unclear. We showed that homozygous deletions in the *STRC* region were identified in 17 of 398 (4.3%) mild-to-moderate SNHL subjects, and our results suggested that *STRC* deletions were the second most common cause of mild-to-moderate SNHL after SNVs in *GJB2*.

The *STRC* gene encodes the large extracellular structural protein stereocilin, which is expressed in the outer hair cells of the inner ear. Stereocilin is associated with the formation of tip link connectors between stereocilia and connections between outer hair cells and the tectorial membrane. Stereocilin-null mutant mice show progressive hearing loss, and their hair bundle stiffness appears decreased despite no apparent structural abnormalities^18–20^. In the present study, we observed that hearing loss did not progress to a severe level in the patients even after 50 years of age. SNHL appearing at birth is detectable via NHS; however, only 7 of 17 subjects were diagnosed with hearing loss through NHS. Indeed, NHS has been implemented since the year 2000 in Japan. NHS is not required by the law, and is provided based on the parents’ request. Therefore, we inferred that hearing loss could have been detected in all cases if NHS was universally applicable.

One region of chromosome 15q15.3 contains the *STRC* gene and the pseudo-*STRC* (*pSTRC*) gene, which has 98% homology to the functional *STRC* gene. The *pSTRC* gene arose in a segmental duplication with other genes (e.g., *CATSPER2* located 100 kb downstream on chromosome 15q15.3). *CATSPER2* is involved in sperm motility, and is responsible for driving the hyperactivated motility that is essential for fertilization^10^. Zhang *et al*. reported three unrelated Iranian families with SNHL and male infertility caused by a long deletion involving four genes (*KIAA0377*, *CKMT1B*, *STRC*, and *CATSPER2*) at chromosome 15q15.3^9^. In the present study, we found a homozygous long deletion involving the *STRC* and *CATSPER2* genes in 15 of 17 cases (88.2%). Consequently, patients with a *STRC* deletion might also possess a *CATSPER2* deletion that could be involved in hearing loss and male infertility. This is important information that should be shared during genetic counseling.

In summary, we performed CNV analysis in *STRC* using NGS read data from the platform used for the current social health insurance-based genetic testing in Japan as a first screening step, followed by confirmation using custom aCGH. We clarified the prevalence of causative *STRC* deletions in a Japanese population, and our data indicate that these were the second most common cause of mild-to-moderate hearing loss, which was congenital and non-progressive in the population.

## Supplementary information

Supplementary information

## Data Availability

The datasets used and/or analyzed during the current study are available from the corresponding author on reasonable request.
