# Supplementary material for: Frequency and clinical features of hearing loss caused by STRC deletions
Source: Sci Rep. 2019 Mar 13;9:4408. doi: 10.1038/s41598-019-40586-7 (PMC6416315; doi:10.1038/s41598-019-40586-7)
Supplement: Supplementary file 1 — Supplementary information [file 41598_2019_40586_MOESM1_ESM.pdf]

Supplementary Information

Frequency and clinical features of hearing loss caused by *STRC* deletions

Yoh Yokota, Hideaki Moteki, Shin-ya Nishio, Tomomi Yamaguchi, Keiko Wakui, Yumiko Kobayashi, Kenji Ohyama, Hiromitsu Miyazaki, Rina Matsuoka, Satoko Abe, Kozo Kumakawa, Masahiro Takahashi, Hirofumi Sakaguchi, Natsumi Uehara, Takashi Ishino, Tomoki Kosho, Yoshimitsu Fukushima, Shin-ichi Usami

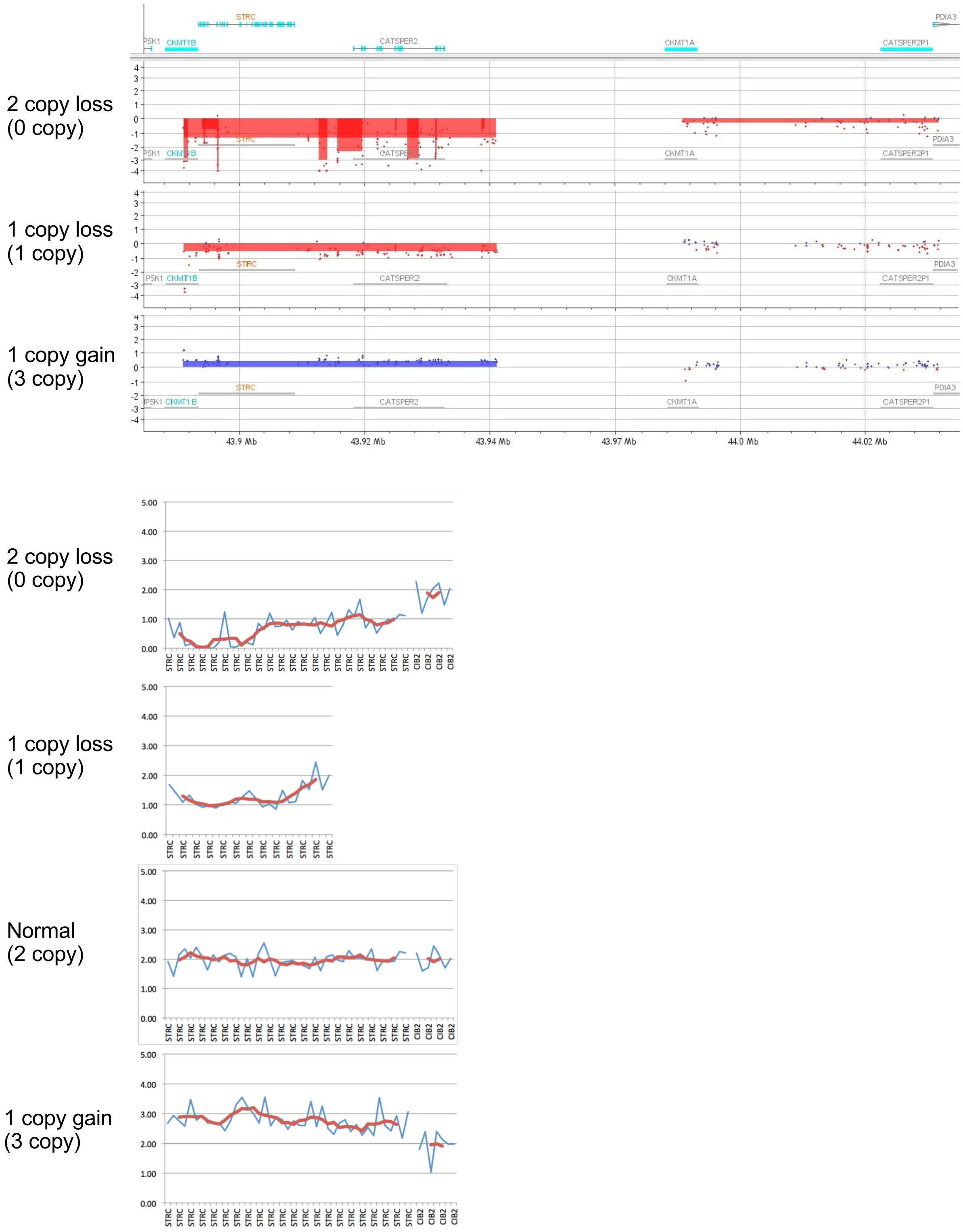

Figure S1. The results of custom array comparative genomic hybridization (aCGH) of the samples that had a 2-copy loss (0 copy), 1-copy loss (1 copy), or 1-copy gain (3 copy), and the results of copy number analysis with the next-generation sequencing (NGS) dataset used in the present study. There was a high concordance between the CNV analysis with the NGS dataset and aCGH in terms of the number of gene copies in both heterozygous and homozygous cases.

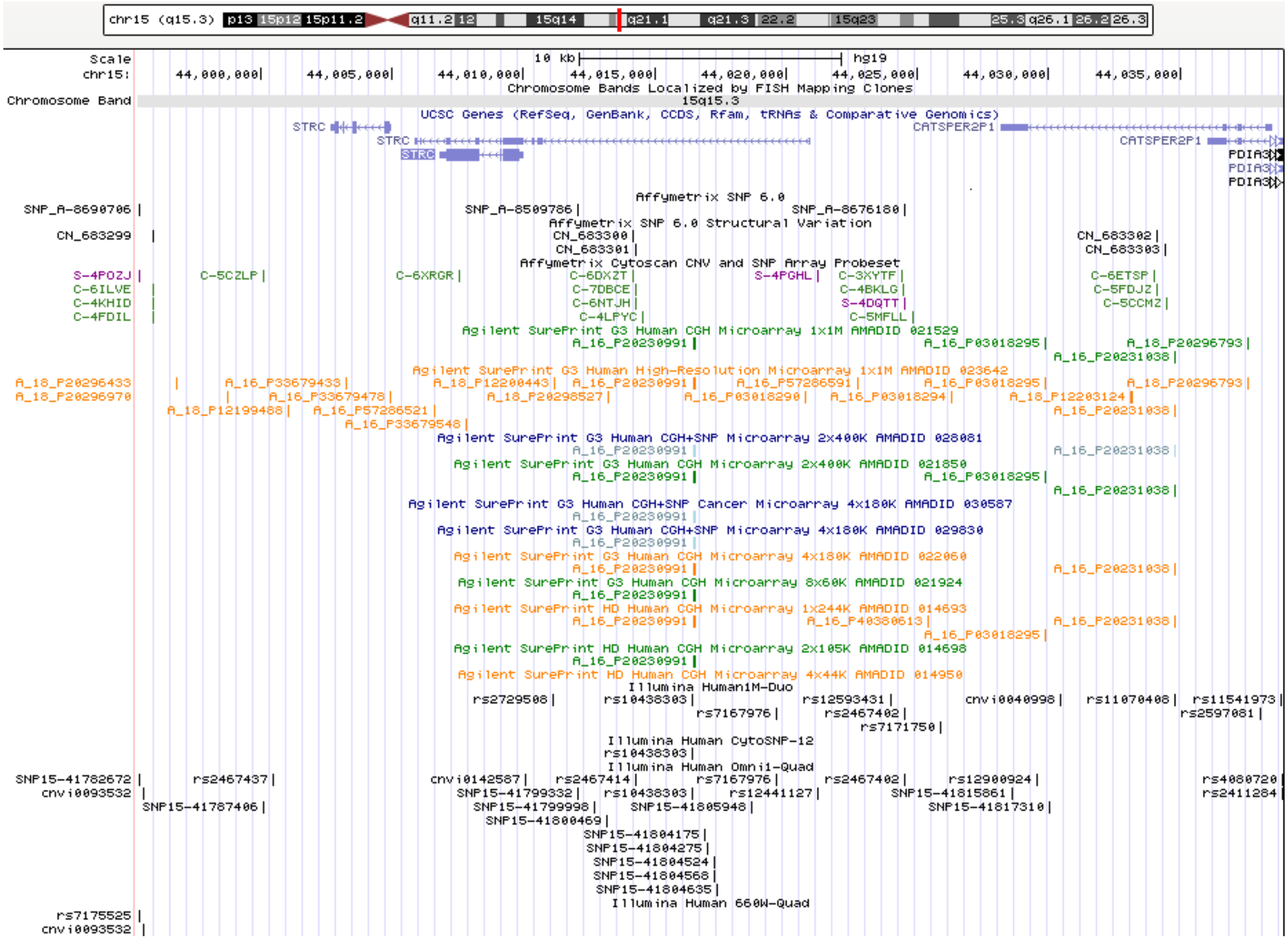

Figure S2. Depiction of probe density in commercially available arrays in the UCSC genome browser. For the *STRC* gene and its surrounding region, only a few probes lay across the commercially available whole-genome CGH array.

Table S1. 68 deafness-causative genes.

Number	Locus Symbol	Gene Symbol	Gene ID	Transcript Variant
1	DFNA1	*DIAPH1*	NM_005219	NM_001079812
2	DFNA2	*KCNQ4*	NM_004700	NM_172163
3	DFNA2	*GJB3*	NM_024009	NM_001005752
4	DFNA3	*GJB6*	NM_006783	NM_001110219
				NM_001110220
				NM_001110221
5	DFNA4	*MYH14*	NM_024729	NM_001145809.
				NM_001077186
6	DFNA5	*DFNA5*	NM_004403	NM_001127454
				NM_001127453
7	DFNA6/14/38	*WFS1*	NM_006005	NM_001145853
8	DFNA8/12/DFNB21	*TECTA*	NM_005422	-
9	DFNA9/31	*COCH*	NM_004086	NM_001135058
10	DFNA10	*EYA4*	NM_004100	NM_172103
				NM_172105
11	DFNA11/DFNB2/USH1B	*MYO7A*	NM_000260	NM_001127179
				NM_001127180
12	DFNA13/DFNB53/STL3	*COL11A2*	NM_080680	NM_080679
				NM_080681
				NM_001163771
13	DFNA15	*POU4F3*	NM_002700	-
14	DFNA17	*MYH9*	NM_002473	-
15	DFNA20/DFNA26	*ACTG1*	NM_001614	NM_001199954
16	DFNA22/DFNB37	*MYO6*	NM_004999	-
17	DFNA25	*SLC17A8*	NM_139319	NM_001145288
18	DFNA28	*GRHL2*	NM_024915	-
19	DFNA36/DFNB7/11	*TMC1*	NM_138691	-
20	DFNA40	*CRYM*	NM_001888	NM_001014444
21	DFNA44	*CCDC50*	NM_178335	NM_174908
22	DFNA48	*MYO1A*	NM_005379	-
23	DFNA50	*MIRN96*		
24	DFNA51	*TJP2*	NM_001170414	NM_004817
				NM_201629
				NM_001170630
				NM_001170415
				NM_001170416
25	DFNA64	*DIABLO*	NM_019887	NM_138929
26		*CEACAM16*	NM_001039213	-
27	DFNB1/DFNA3	*GJB2*	NM_004004	-
28	DFNB3	*MYO15A*	NM_016239	-
29	DFNB4/Pendred	*SLC26A4*	NM_000441	-
30	DFNB6	*TMIE*	NM_147196	-
31	DFNB8/10	*TMPRSS3*	NM_024022	NM_032405
32	DFNB9	*OTOF*	NM_194248	NM_194322
				NM_194323
				NM_004802
33	DFNB12/USH1D	*CDH23*	NM_022124	NM_001171930
				NM_001171931
				NM_001171932
				NM_001171933
				NM_001171934
				NM_001171936
				NM_052836
34	DFNB15/72/95	*GIPC3*	NM_133261	-
35	DFNB16	*STRC*	NM_153700	-
36	DFNB18/USH1C	*USH1C*	NM_153676	NM_005709
37	DFNB22	*OTOA*	NM_144672	NM_001161683
				NM_170664
38	DFNB23/USH1F	*PCDH15*	NM_033056	NM_001142763

				NM_001142764<br>NM_001142765<br>NM_001142766<br>NM_001142767<br>NM_001142769<br>NM_001142770<br>NM_001142771<br>NM_001142772<br>NM_001142773
39	DFNB24	*RDX*	NM_002906	-
40	DFNB25	*GRXCR1*	NM_001080476	-
41	DFNB28	*TRIOBP*	NM_007032	NM_138632<br>NM_001039141
42	DFNB29	*CLDN14*	NM_144492	NM_001146077<br>NM_001146078<br>NM_001146079<br>NM_012130
43	DFNB30	*MYO3A*	NM_017433	-
44	DFNB31/USH2D	*WHRN*	NM_015404	NM_001083885<br>NM_001173425
45	DFNB35	*ESRRB*	NM_004452	-
46	DFNB36	*ESPN*	NM_031475	-
47	DFNB39	*HGF*	NM_000601	NM_001010931<br>NM_001010932<br>NM_001010933<br>NM_001010934
48	DFNB42	*ILDR1*	NM_001199799	NM_001199800<br>NM_175924
49	DFNB48	*CIB2*	NM_006383	NM_001271888<br>NM_001271889
50	DFNB49	*MARVELD2*	NM_001038603	NM_001244734
51	DFNB59	*DFNB59*	NM_001042702	-
52	DFNB61	*SLC26A5*	NM_206883	NM_001167962<br>NM_206884<br>NM_206885<br>NM_198999
53	DFNB63	*LRTOMT*	NM_001145307	NM_001145308<br>NM_001205138<br>NM_145309
54	DFNB66/67	*LHFPL5*	NM_182548	-
55	DFNB70	*PNPT1*	NM_033109	-
56	DFNB74	*MSRB3*	NM_198080	NM_001031679<br>NM_001193460<br>NM_001193461
57	DFNB77	*LOXHD1*	NM_144612	NM_001173129<br>NM_001145472<br>NM_001145473
58	DFNB79	*TPRN*	NM_001128228	-
59	DFNB82	*GPSM2*	NM_013296	-
60	DFNB84	*PTPRQ*	NM_001145026	-
61	DFNB89	*KARS*	NM_005548	NM_001130089
62	DFNB91	*SERPINB6*	NM_004568	NM_001195291
63	DFNB93	*CABP2*	NM_016366	-
64	USH2A	*USH2A*	NM_007123	NM_206933
65	DFNX1	*PRPS1*	NM_002764	NM_001204402
66	DFNX2	*POU3F4*	NM_000307	-
67	DFNX4	*SMPX*	NM_014332	-
68	DFNX6	*COL4A6*	NM_001847	NM_033641
